# Increasing vegetable intakes: rationale and systematic review of published interventions

**DOI:** 10.1007/s00394-015-1130-8

**Published:** 2016-01-11

**Authors:** Katherine M. Appleton, Ann Hemingway, Laure Saulais, Caterina Dinnella, Erminio Monteleone, Laurence Depezay, David Morizet, F. J. Armando Perez-Cueto, Ann Bevan, Heather Hartwell

**Affiliations:** Research Centre for Behaviour Change, Department of Psychology, Faculty of Science and Technology, Bournemouth University, Poole House, Fern Barrow, Poole, Dorset BH12 5BB UK; Faculty of Health and Social Sciences, Bournemouth University, Bournemouth, Dorset UK; Centre for Food and Hospitality Research, Institut Paul Bocuse, Ecully, France; Department of the Management of Agriculture, Forestry and Food Systems, University of Firenze, Florence, Italy; Food and Behaviours Department, Corporate Research and Communication, Bonduelle, Villeneuve D’Ascq, France; Department of Food Science, University of Copenhagen, Copenhagen, Denmark; Faculty of Management, Bournemouth University, Poole, Dorset UK

**Keywords:** Vegetables, Interventions, Systematic review, Published literature

## Abstract

**Purpose:**

While the health benefits of a high fruit and vegetable consumption are well known and considerable work has attempted to improve intakes, increasing evidence also recognises a distinction between fruit and vegetables, both in their impacts on health and in consumption patterns. Increasing work suggests health benefits from a high consumption specifically of vegetables, yet intakes remain low, and barriers to increasing intakes are prevalent making intervention difficult. A systematic review was undertaken to identify from the published literature all studies reporting an intervention to increase intakes of vegetables as a distinct food group.

**Methods:**

Databases—PubMed, PsychInfo and Medline—were searched over all years of records until April 2015 using pre-specified terms.

**Results:**

Our searches identified 77 studies, detailing 140 interventions, of which 133 (81 %) interventions were conducted in children. Interventions aimed to use or change hedonic factors, such as taste, liking and familiarity (*n* = 72), use or change environmental factors (*n* = 39), use or change cognitive factors (*n* = 19), or a combination of strategies (*n* = 10). Increased vegetable acceptance, selection and/or consumption were reported to some degree in 116 (83 %) interventions, but the majority of effects seem small and inconsistent.

**Conclusions:**

Greater percent success is currently found from environmental, educational and multi-component interventions, but publication bias is likely, and long-term effects and cost-effectiveness are rarely considered. A focus on long-term benefits and sustained behaviour change is required. Certain population groups are also noticeably absent from the current list of tried interventions.

## Introduction

### Health benefits of high fruit and vegetable intakes

The health benefits of a high consumption of fruits and vegetables are well known [1]. Associations with all-cause mortality [2, 3] and mortality from cardiovascular disease [2, 3], including coronary heart disease [4] and stroke [5, 6], are well evidenced. Associations also suggest a reduced risk of hypertension [7], osteoporosis [8], body weight and adiposity [9, 10], dementia and cognitive decline [11, 12], and some cancers [13–15], although the evidence for cancers is less consistent [1, 3]. Intervention studies increasing the consumption of fruits and vegetables also demonstrate improved microvascular function [16], improved microvascular function and inflammatory status [17], improved profiles in inflammatory and oxidative stress [18], improved immune response [19], and improved weight maintenance [20].

### Consideration of fruits and vegetables as different food groups

However, while fruits and vegetables share health benefits as a result of the provision and interaction of a number of bioactive compounds, including vitamins, minerals, antioxidants, carotenoids and flavenoids [21, 22], the specific bioactive compounds in fruits and in vegetables can vary greatly [21–25]. Their contribution to other dietary features also vary. Fruits typically contain greater dietary sugars, with potential negative impacts on both health and on public willingness to consume them [22, 26]. Vegetables, by comparison, can contain more protein and fibre [22] and are more often processed prior to consumption. This processing can both increase and decrease micronutrient bioavailability and activity, again impacting on health benefits [22, 25, 27–31]. Many studies that separate fruits and vegetables find different effects of the different food classes on health outcomes [2, 12, 32–35]. These differences between fruits and vegetables argue for the consideration of fruits and vegetables, in terms of health, as separate and different food types.

While differing in their potential health benefits, fruits and vegetables also taste very different, are generally of a different texture and are typically consumed in different manners [36–39]. Fruit is generally sweet, is typically softer in texture, is more often consumed raw, is more frequently consumed and is generally considered more acceptable as a snack, as a drink or as dessert [36–39]. Vegetables, by comparison, can taste bitter, are generally harder in texture, are more often cooked, are more typically consumed and considered more acceptable as part of a meal [36–39], and thus are also more often consumed with other foods as opposed to alone [40]. These different consumption patterns suggest that fruit and vegetable consumption may be differentially determined. Glasson et al. [41] directly compared the determinants of fruit consumption and vegetable consumption in an Australian population, to find fruit consumption to be largely prevented by cost, food preferences, quality, availability and wastage concerns, while vegetable consumption was more frequently prevented by food preferences, lack of time, cost and taste. Differential determinants again argue for the consideration of fruits and vegetables, as separate and different food types, and suggest the potential need for different intervention strategies for increasing fruit and for increasing vegetable consumption.

Furthermore, while population levels of both fruit and vegetable consumption remain below World Health Organization recommendations across the world [42, 43], interventions to increase fruit and vegetable intakes more often target fruit, and typically report greater success for fruit consumption compared to that for vegetables, for both children and adults [44–46]. These findings suggest not only a need for different intervention strategies for increasing fruit and increasing vegetable consumption, but a real need for strategies that achieve successful increases in vegetable-specific consumption. This paper focuses specifically on vegetable consumption.

## Vegetable-specific consumption

### Health benefits of high vegetable-specific intakes

Various studies demonstrate health benefits from a high consumption specifically of vegetables (i.e. from vegetables alone, as opposed to in combination with fruits, as occurs when considering fruits and vegetables together). Observational studies demonstrate reduced risk of cardiovascular disease [2], type II diabetes [34], non-gallstone-related acute pancreatitis [33], various cancers [2, 47–50] and cognitive decline [32]. Meta-analyses of observational studies demonstrate associations between a higher vegetable consumption and reduced risk of stroke [6], dementia and cognitive decline [12], and from various cancers [14, 15, 35, 51, 52], although again the evidence for cancers is inconsistent. Meta-analyses of prospective studies find no benefits for breast cancer risk [53], gastric cancer risk [54], pancreatic cancer risk [55] and bladder cancer risk [56]. Meta-analyses also report no benefits of overall vegetable consumption for type II diabetes [57–59], although dose–response meta-analyses also suggest benefit up to 2–3 servings/day and a threshold beyond this where type II diabetes risk does not reduce further [60].

Specific vegetable groups or types of vegetables have also been associated with improved health outcomes. Intakes of dark green leafy vegetables have been associated with reduced risk for type II diabetes [57–59], reduced risk for a number of cancers [48, 49, 61] and with reduced depression [62]. High intakes of cruciferous vegetables have been associated with reduced risk from various cancers [63–70]. Intakes of beta-carotene-rich vegetables, yellow- and red-pigmented vegetables, and fruiting vegetables have also been associated with reduced risk from various cancers [48–50, 61], and root vegetable consumption has been associated with reduced type II diabetes risk [60].

Much of this evidence, however, stems from limited studies, and the body of evidence is far from conclusive [64, 66]. Prospective and cross-sectional studies are easily criticised for potential confounding, case control comparisons may suffer from bias towards differences between groups due to comparisons between cases and health conscious (and consequently) healthy controls [71], and study designs do not allow determination of causality. Considerable further work is required before conclusions can be drawn. The majority of studies investigating effects of vegetable consumption, furthermore, do not investigate vegetable consumption independent of fruit consumption or other aspects of the diet. While fruits and vegetables are frequently consumed together, associations may reflect not just associations with vegetables, but associations with produce consumption in general, or with a healthier diet/lifestyle [1, 72]. A recent systematic review by Fulton et al. [72] reports impacts for fruit and vegetable interventions not only from micronutrient intakes but also from changes to the whole dietary profile. Lifestyle factors are frequently included in studies as confounders, but it is often difficult to control for all potential confounders, and adjustment for other dietary aspects, particularly fruit consumption, is less common. Associations will also depend on the definition of vegetables used, and the inclusion or not of certain vegetables in certain categories. Potatoes, for example, are sometimes included among vegetables, sometimes included as ‘white’ vegetables, and sometimes not considered at all [21, 22].

Thus, for improved health, increasing intakes of vegetables are required. For intakes of vegetables to be increased, strategies and interventions are needed. These interventions should be based on in-depth understanding of the underlying determinants of low vegetable consumption.

## Determinants of vegetable-specific consumption

Various research has been undertaken to understand the associations with, and reasons for, vegetable consumption, independent of fruit consumption. In young children, the bitter and undesirable taste of vegetables is often provided as a major barrier to vegetable consumption [73–75], and food neophobia (the reluctance to eat, or the avoidance of, novel foods [76]) particularly, can interfere with young children’s acceptance of vegetables [76–78]. This neophobia typically results in the rejection of bitter tasting foods and foods that do not ‘’look right’’ [76], of which vegetables are good examples.

As children age, taste, appearance and liking continue to be important [79], but low vegetable consumption is frequently also associated with various characteristics of the family environment. These factors include low parental education and socio-economic status [80–82], low vegetable consumption by parents and caregivers [83–85], low availability and negative perceptions of vegetables in the home [86] and a family environment that is unsupportive of vegetable consumption [83, 85, 87]. Vegetable consumption is higher, for example, in families where vegetables are disguised or sauces are used to mask undesirable tastes and appearances [79, 83, 85], where vegetables are more often incorporated into composite foods as opposed to consumed alone to dilute negative tastes and appearances [79], where meals are home cooked to accommodate individual preferences [83], and where games are played to encourage vegetable consumption [85]. The expression of neophobic behaviour towards vegetables also appears to be mitigated by high parental education and high socio-economic status [77, 88] and again by a positive and supportive environment [89, 90].

Taste, appearance, liking and the surrounding environment continue to be important as children become adolescents, but individual cognitions also gain increasing importance. Low vegetable consumption in adolescents has again been associated with low parental education and socio-economic status [91], low vegetable consumption by the parents [92], low availability and a family environment that is unsupportive of vegetable consumption [91, 93]. Vegetable consumption in adolescents, however, has also been associated with an awareness of the importance of vegetables for health, and a willingness and ability to ask for vegetables from parents [93].

In adults, higher vegetable consumption has been associated with higher liking for the taste of vegetables [41, 94, 95], higher appreciation of health and the value of a healthy diet [94], greater nutritional and culinary knowledge [96, 97], and with several related food habits and eating practices [98, 99], including usual consumption of meals as opposed to snacks [94, 100], increased time and willingness to prepare and cook home-cooked meals [41, 94, 95, 97], and a low consumption of fast food [94]. The transfer of childhood eating habits and food preferences into adulthood is well known, and adult vegetable intake is often related to childhood experiences [94]. Neophobic tendencies also typically last well into adulthood, and typically correlate negatively with liking for and frequency of vegetable consumption in adulthood [101–103]. The individual preferences of one family member can also have impacts on the rest of the family, with most family units opting to cook only one meal of acceptability to all family members [104, 105]. Given the importance of adult consumption for children, many of the determinants of adult consumption will also impact on child consumption.

Alongside individual preferences, higher vegetable consumption in adults is also related to increased availability [98, 106, 107] and reduced cost [41, 95, 97, 108], and low consumption is largely associated with lower socio-economic status [109, 110], lower income [44, 107], living in a more deprived area or lower income neighbourhood (an indirect measure of socio-economic status) [44] and lower education [109, 111].

Research thus, suggests a variety of reasons behind low vegetable consumption, ranging from taste and pleasure, to individual cognitions and health beliefs, and to aspects beyond the individual including society and the environment. Many of these reasons have been targeted by interventions.

## Strategies to increase vegetable-specific consumption: systematic review

Various reports of strategies to increase vegetable-specific consumption are available. A comprehensive collection of these interventions, and an evaluation of success, however, is currently lacking. The aim of this work was to systematically review the published literature to identify all published interventions aiming to increase vegetable-specific consumption.

## Method

**Fig. 1:**
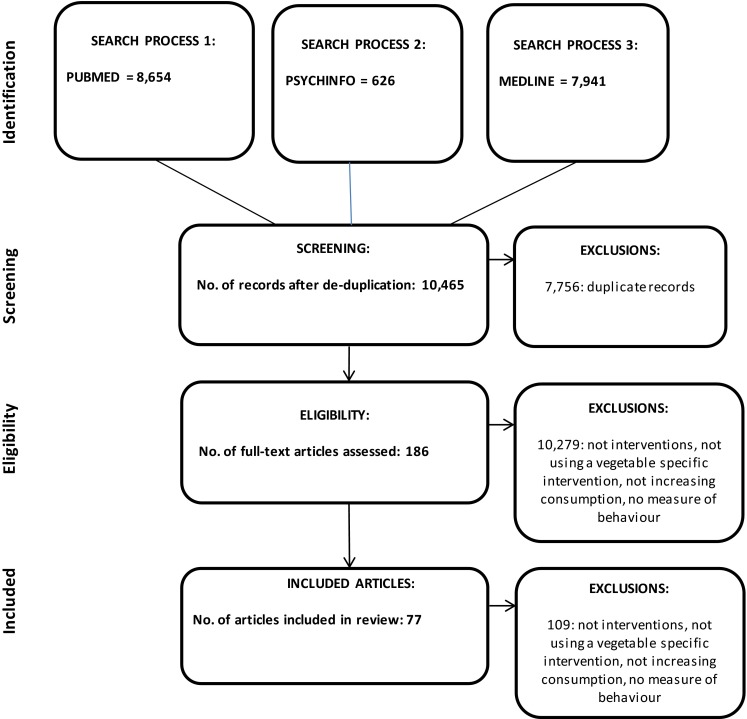
PRISMA diagram showing the results of the search process

The objective of the review was to identify from the published literature all studies reporting an intervention to increase vegetable intakes, where vegetables were considered as a separate and distinct food group, and the intervention focused specifically on increasing intakes of this food group. Three databases: PubMed, PsychInfo, and Medline, were searched over all years of records for all studies with the terms ‘vegetable’ and ‘vegetables’ in the ‘title’. These search criteria were used to limit the search results to studies with a focus on vegetables. No other search criteria and no limits were used. All titles were screened for relevance and then all abstracts. Two review authors independently conducted all searches, screened all titles and screened all abstracts (KMA, AH or HH). Studies were included in the review if they involved an intervention designed primarily to increase vegetable intakes as a specific and distinct food group, and if they intended to change behaviour—vegetable selection, purchasing or vegetable consumption. Studies were not included if they did not include an intervention, if the intervention targeted fruit and vegetable intakes [112], if the intervention targeted vegetables and other foods, e.g. vegetables and wholegrains [113, 114]; if the intervention involved changing consumption as opposed to increasing consumption [115], or if they did not include a measure of behaviour, but instead only measured correlates of behaviour such as intentions, attitudes, and knowledge [116, 117]. Studies measuring tasting were included where tasting was voluntary, where amount tasted was voluntary and where tasting/amount was measured, but studies where tasting was compulsory and/or prespecified, e.g. to make hedonic judgements, were not included [118, 119]. Studies were included regardless of the use or not of a comparison for an intervention, or the type of comparison used. A study using a vegetable-specific intervention that is compared with a fruit-specific intervention, for example, is included [120] (all other criteria were also met). Relevant articles were also searched for other suitable studies. Searches of conference abstracts, book chapters, etc., were not undertaken, thus studies are only included if reported in full articles. Details from all studies were subsequently tabulated by one review author (KMA) and checked by an additional review author (AH or HH). All tables are provided in the “Results” section. No other data were extracted. Due to the early nature of the research area, the limited number of studies available per intervention type, and high heterogeneity between study methodologies, risk of bias was not assessed, and no attempt was made to combine studies, e.g. through meta-analysis. The review was undertaken using PRISMA guidelines and a PRISMA diagram illustrating the outcomes of the review process is given in Fig. 1.

## Results

Searches were most recently conducted on 28 April 2015. The results of the searches are given in the PRISMA diagram in Fig. 1. A total of 77 studies were identified, reporting the impacts of 140 interventions. Details of all studies are presented by intervention type in Tables 1, 2, 3, 4, 5 and 6. Interventions have been broadly classified as those focussing on hedonic determinants of vegetable intake, such as taste, familiarity and liking—Tables 1, 2 and 3, those focussing on environmental determinants of vegetable intake—Table 4, those focussing on cognitive determinants—Table 5, and those using a combination of approaches—Table 6. Studies reporting two or more different interventions are included separately in separate tables, where appropriate. Within each table, studies are ordered by age of target audience. Of the interventions identified, 113 (81 %) interventions focus on improving intakes in children. Early intervention will maximize health benefits [45], and eating habits in childhood are likely to extend into adulthood [75, 94].

### Interventions aiming to change or use hedonic factors

Eleven interventions focus on changing or using the taste or familiarity of a vegetable/vegetable product on a single occasion (Table 1). Six of these interventions suggest that the addition of a liked taste or flavour in the form of salt [121, 123] and in the form of a flavoured dip [122] or condiments [125] can increase vegetable consumption on a single occasion. Guidelines regarding salt intake and the possible impact of increasing preferences for salty flavours must also be considered, but these studies demonstrate a potentially useful strategy. The addition of fat to a vegetable product did not result in increased intakes [121], but increased intakes were found following the use of familiar as opposed to novel vegetables products [124].

**Table 1:** Published interventions utilising taste or familiarity on a single occasion to increase vegetable intake

Reference/intervention	Aim	Intervention	Comparison	Results	Conclusions
Bouhlal et al. [121] Taste	To increase V intakes in children aged 18–37 months (*n* = 74)	1. Reduction in salt provision (0 %) 2. Increase in salt provision (1.2 %)	Usual salt provision (0.6 %)	Less V consumed in I1 vs C. No effects for I2	Salt addition should be limited, but its suppression in V, whose intake is to be promoted, should be considered cautiously
Bouhlal et al. [121] Taste	To increase V intakes in children aged 18–37 months (*n* = 74)	1. Reduction in fat provision (0 %) 2. Increase in fat provision (5 %)	Usual fat provision (2.5 %)	No differences between conditions	Fat addition could be avoided in foods for children without having an impact on palatability
Savage et al. [122] Study 1 Taste	To increase willingness to taste, liking and consumption of V in children aged 3–5 years (*n* = 34)	Single exposure to 3 target V paired with: 1. Plain reduced-fat dip 2. Favoured flavoured reduced-fat dip	Single exposure to 3 target V paired with no dip	Increased willingness to taste in I vs C. No differences between I1 and I2	Offering V with reduced-fat dips containing familiar flavours can increase tasting and thereby promote liking, acceptance and consumption of V, including V previously rejected or disliked
Savage et al. [122] Study 2 Taste	To increase willingness to taste, liking and consumption of V in children aged 3–5 years (*n* = 26/27)	Single exposure to 2 target unfamiliar or disliked V (celery, squash) with a favoured flavoured reduced-fat dip	Single exposure to 2 target V with no dip	Increased intakes in I vs C	Offering V with reduced-fat dips containing familiar flavours can increase tasting and thereby promote liking, acceptance and consumption of V, including V previously rejected or disliked
Bouhlal et al. [123] Taste	To increase V intakes in children aged 8–11 years (*n* = 75)	1. Reduction in salt provision (0 %) 2. Increase in salt provision (1.2 %)	Usual salt provision (0.6 %)	Less V consumed in I1 vs C. No effects for I2	Salt content has a positive and food-specific effect on intake
Morizet et al. [124] Familiarity	To increase V dish selection in 8–11 year olds. School-based intervention	Novel V dishes with no label (carrots *n* = 60, broccoli *n* = 65)	Familiar V dishes with no label	Increased selection for C vs I	Adding a label with the V name can increase children’s willingness to select a novel V dish instead of a familiar one. Familiar V are otherwise more likely to be consumed
Ahearn [125] Taste	To increase V consumption in a 14 year old boy with autism. Case study	Simultaneous presentation of 3 V with liked condiments	No control	I significantly increased intakes	Adding condiments increased food acceptance across three food items

Interventions ordered by age of target audience

*C* comparison, *I* intervention, *V* vegetable, *vs* versus

Fifty-two interventions focus on increasing liking and familiarity with repeated experience. These interventions use learning techniques, including repeated exposure (*n* = 23), ensuring that exposure is positive via pairing with liked flavours (*n* = 14), pairing with beneficial nutrients (*n* = 6), pairing with external reinforcement (*n* = 7), the use of positive models (*n* = 1) and the use of reinforcement plus models (*n* = 1) (Table 2). Many of these interventions demonstrate success by improving liking and/or consumption: 16 of 23 (70 %) using repeated exposure; 12 of 14 (86 %) using pairing with liked flavours; four of six (67 %) using pairing with nutrients; all seven (100 %) using pairing with reinforcement; and the one (100 %) using reinforcement plus modelling. Effects, however, are far from robust or consistent (i.e. effects are often found in one measure, but not in others), are often small, and tend to be limited to the specific vegetable used during exposure. Conditions within studies, furthermore, are often confounded, making mechanisms difficult to elucidate. In many studies that purport to investigate exposure, the exposure is in combination with other food components [129], modelling [75] or rewards in the form of praise or other positive interactions [139], thus effects may in fact occur partly due to conditioning. In many studies that purport to measure conditioning, exposure is not controlled for [136, 151]. Many of these interventions furthermore also involve children’s parents, and so may have benefits not just by allowing tasting and experience for the child, but also by improving parental perceptions of vegetables, improving attitudes towards vegetables in the home, and improving parental education and knowledge [75]. Interestingly, some of the interventions included in Table 2 report parental opinions of the intervention [75, 129], but as far as we can tell, none specifically tested parental knowledge or education as a result of the intervention for their children.

**Table 2:** Published interventions utilising learning (exposure, associative conditioning, instrumental conditioning or modelling) to increase vegetable intake

Reference/intervention	Aim	Intervention	Comparison	Results	Conclusions
Remy et al. [126] Exposure Conditioning	To increase V acceptance at complementary feeding (children aged 4–8 months)	1. RE—10 exposures to target V puree (*n* = 32) 2. FFL—10 exposures to target V puree paired with sweetener (*n* = 32) 3. FNL—10 exposures to target V puree paired with energy (*n* = 31)	Control V—limited experimental exposures	No differences in intake in I vs C postintervention. Intake of target V puree increased postintervention and at 3-m follow-up in I1 and I2, but not I3 vs pretest. Liking increased only in I1. No effects at 6-m follow-up	RE is as effective as and simpler to implement than FFL and more effective than FNL for increasing V acceptance at complementary feeding
Mennella et al. [127] Study 2 Exposure	To increase V acceptance in infants aged 4–9 months	1. 8-day exposure to single V (*n* = 11) 2. 8-day exposure to several V in different meals (*n* = 12) 3. 8-day exposure to several V in the same meals (*n* = 12)	No control (pre-post comparison only)	Increased intakes of green beans, carrots and spinach in I3 compared to before. Trend towards increased intakes of green beans in I1 and I2 after intervention vs before	Repeated opportunities to taste a particular or a variety of foods may promote willingness to eat V
Mennella et al. [128] Exposure	To increase V acceptance in infants aged 6–11 months	Milk formula flavoured with hydrolysate (similar taste to target V) (*n* = 24)	Milk formula (no hydrolysate taste) (*n* = 50)	Less target V (and relative to other V) was consumed by I vs C	Taste preferences are initially specific to the context they are learnt (in this case milk)
Hetherington et al. [129] Exposure	To increase liking and acceptance of target V and unfamiliar V during weaning (children aged 6–12 months)	12 daily exposures to target V puree added to milk, then 12 × twice daily exposures to target V puree added to baby rice (*n* = 18)	Plain milk for 12 days, then plain rice for 12 days (*n* = 18)	Intake, liking and eating pace were greater for target V but not unfamiliar V for I vs C, at end of intervention No differences at 6 or 18-m follow-up	Early exposure to vegetables in a step-by-step method could be included in complimentary feeding guidelines to enhance V intakes
Maier et al. [130] Exposure	To increase V acceptance in children aged 7 months (*n* = 49)	1. 8 daily exposures to disliked V 2. 8 daily exposures to liked V	No control (pre-post comparison only)	Intakes increased in I1 and I2, greater increases for I1. Effects sustained for 9 months (self-report)	When a V is initially disliked it is worth persisting in feeding (exposure) for at least 8 subsequent meals
Caton et al. [131] Exposure Conditioning	To increase V acceptance in children aged 9–38 months	1. RE—10 exposures to target V puree (*n* = 22) 2. FFL—10 exposures to target V puree paired with sweetener (*n* = 25) 3. FNL—10 exposures to target V puree paired with energy (*n* = 25)	Control V—limited experimental exposures	Greater intake in I vs C, postintervention and 5-w follow-up. No differences between interventions postintervention. Higher intakes in I1 vs I2 at 5-w follow-up	RE, FFL and FNL were effective for increasing V acceptance, and equally so
Barends et al. [120] Exposure	To increase V intakes in children aged 12–23 months	Started weaning with target V (green beans/artichoke), exclusive V for 18 days, 9 exposures to target V (*n* = 51)	Starting weaning with fruit, exclusive fruit for 18 days, 9 exposures to target fruit (*n* = 50)	Greater liking and intake of green beans, and greater intake of novel V, postintervention in I vs C, but no effects for artichoke	Weaning with V but not with fruits, may promote V acceptance in children
Barends et al. [132]	Follow-up of Barends et al. [120]	As above	As above	Greater reported liking and daily intake of V at 12 months in I vs C, but no differences at 23 months. No differences in measured intake at either time point	Weaning exclusively with V results in a higher daily V consumption until at least 12 months of age
Ahern et al. [133] Exposure Conditioning	To increase V acceptance in children aged 12–60 months (*n* = 29)	1. RE—8 exposures to target V puree 2. FFL—8 exposures to target V puree paired with apple puree for sweetness	Control V—0 exposures	Significant increases in V intake from pre- to postintervention. No differences between conditions	No effects of exposure The addition of a familiar flavour (FFL learning) confers no advantage above exposure
Hausner et al. [134] Exposure Conditioning	To increase V acceptance in children aged 22–38 months	1. RE—10 exposures to target V puree (*n* = 32) 2. FFL—10 exposures to target V puree paired with sweetener (*n* = 33) 3. FNL—10 exposures to target V puree paired with energy (*n* = 39)	Control V—limited experimental exposures	Greater intake in I1 and I2 vs pretest, at postintervention, 3-m and 6-m follow-up. No effects in I3. No comparison with C	RE and FFL were effective for increasing V acceptance, and equally so. FNL was not effective
Bouhlal et al. [135] Exposure Conditioning	To increase V acceptance in children aged 2–3 years	1. RE—8 exposures to target V puree (*n* = 47) 2. FFL—8 exposures to target V puree paired with salt (*n* = 54) 3. FFL—8 exposures to target V puree paired with spice (*n* = 50)	Control V—no exposures	Greater intake in all I vs pretest, at postintervention, 1-, 3- and 6-m follow-up. Greater effects in I1 vs I2 and I3. Increases in liking also in I1 and I2. No change in C, but no statistical comparison with I provided	RE appears to be the simplest choice to increase V intake in the short and long term in toddlers
De Wild et al. [136] Conditioning	To increase preferences and intake for target V in children aged 2–4 years (*n* = 28)	Seven twice weekly consumptions of target V soup (endive/spinach) paired with high energy	Seven twice weekly consumptions of target V soup (spinach/endive) paired with low energy	Increased preferences for I vs C immediately after intervention, no effects on intake. No effects at 2 and 6-month follow-up. Increase in intake for all V from pre- to postintervention	Results show an effect of exposure on intake, but not conditioning. Effects of conditioning are found in preferences
Holley et al. [137] Exposure Conditioning Modelling	To increase acceptance of a disliked V in children aged 2–4 years	1. Exposure—daily exposure to target V for 14 days (*n* = 29) 2. Exposure + modelling—parent also consumed target V and gave positive comment for 14 days (*n* = 27) 3. Exposure + rewards—child given praise and non-food reward for tasting target V for 14 days (*n* = 29) 4. Exposure, modelling + rewards—all above strategies for 14 days (*n* = 27)	No exposure or other intervention	No differences between I and C when all groups analysed together. Significant increases in V intake and liking in I3 and I4 vs C in secondary analyses	Parent-led interventions based around modelling and offering incentives may present cost-efficient ways to increase children’s V consumption
Wardle et al. [75] Exposure	To increase liking and intake for a previously disliked V in children aged 2–6 years. Home-based intervention	Exposure—child given a daily taste of V for 14 days (*n* = 50)	No intervention (wait list) (*n* = 45)	Greater increases in liking, ranking and consumption of V from pre- to postintervention in I vs C	A parent-led, exposure-based intervention involving daily tasting of a V holds promise for improving children’s acceptance of and increasing liking for a previously disliked V
Fildes et al. [138] Exposure Rewards	To increase V acceptance in children aged 3 years. Mailed intervention	Mailed instructions to offer children 14 daily tastes of a disliked V and sticker reward (*n* = 196)	No intervention (usual practice) (*n* = 246)	Intake and liking of V increased in I vs C. Acceptability of the protocol was also very high among I parents	Mailed instructions for taste exposure were effective in increasing children’s acceptance of an initially disliked vegetable
Anzman-Frasca et al. [139] Study 1 Exposure Conditioning	To increase V liking and intakes in children aged 3–6 years (*n* = 41)	1. RE—twice weekly exposures to initially not-liked V for four weeks 2. AC—twice weekly exposures to initially not-liked V with a liked dip for four weeks	No exposure	Liking increased in I1 and I2, vs C, but no differences between I1 and I2	Administering few small tastes of V that are initially not liked, both with and without dip, can have a lasting impact on liking and intake of those V
Anzman-Frasca et al. [139] Study 2 Conditioning	To increase V liking and intakes in children aged 3–6 years (*n* = 43)	AC—twice weekly exposures to initially not-liked V with a liked dip for four weeks	RE—twice weekly exposures to initially not-liked V for four weeks	Liking increased in I and C, but no differences between them	Administering few small tastes of V that are initially not liked, both with and without dip, can have a lasting impact on liking and intake of those V
O’Connell et al. [140] Exposure	To increase V intakes in children aged 3–6 years old	10 exposures of 3 different V at lunch over 30 days (30 exposures) (*n* = 50)	No exposure (*n* = 50)	No differences between conditions	Research should explore the conditions necessary for exposure to increase V intakes in preschool settings
Correia et al. [141] Conditioning	To increase V intakes and willingness to try in pre-school children (3–5 years)	Lunch Target V paired with familiar well-liked food (*n* = 43)	Lunch Target V not paired with familiar well-liked food	No differences in intakes. Willingness to try increased marginally in I vs C	Further research should explore the strategy of pairing vegetables with liked foods
Fisher et al. [142] Conditioning	To increase liking and intakes in bitter-sensitive and insensitive preschoolers	13 exposures to moderately liked V over 7 weeks with: 1. regular salad dressing as dip (*n* = 39) 2. light salad dressing as dip (*n* = 36) 3. regular dressing as sauce (*n* = 38)	13 exposures to moderately liked V over 7 weeks with: no dressing (*n* = 39)	No effects on intake in insensitive children. Higher liking and intakes in bitter-sensitive children in all I vs C. Effects vary based on whether regular or light dressing was provided as a dip or sauce	Offering dips can promote vegetable intake among some children who are sensitive to bitter tastes
Havermans and Jansen [143] Conditioning	To increase liking and preference for a target V taste in children aged 4–6 years (*n* = 13)	6 conditioning trials—V juice paired with sweet taste	6 trials—different V juice not paired with sweet taste	Increase in liking and preference for I vs C	Flavour-flavour learning may be beneficial in increasing children’s liking and acceptance of vegetables
Hendy et al. [144] Rewards	To increase (fruit and) V intakes in 1st, 2nd and 4th graders. School-based intervention	Rewards given for consumption of V at 12 meals (*n* = 96)	Rewards given for consumption of fruit at 12 meals (*n* = 96)	Increased intakes of V following I vs C. Increased preferences for V (marginal) after intervention vs before	The use of rewards as in the Kids Choice programme shows promise as a simple and effective method to increase children’s (fruit and) V acceptance
Cooke et al. [145] Exposure Rewards	To increase V likings and intakes in children aged 4–6 years	Twelve daily taste exposures to target (disliked) V with 1. tangible reward (*n* = 99) 2. social reward (*n* = 106) 3. no reward (exposure only) (*n* = 105)	No exposure (*n* = 112)	Liking increased in I1, I2, and I3 vs C, postintervention and 1- and 3-m follow-up. No differences between interventions. Consumption increased in I1 and I2 vs C postintervention and 1- and 3-m follow-up. Consumption increased in I3 vs C postintervention and 1-m follow-up only	Rewarding children for tasting an initially disliked V produced sustained increases in acceptance, with no negative effects on liking
Corsini et al. [146] Exposure Rewards	To increase liking and consumption of a disliked V in children aged 4–6 years	1. EO—Daily exposure for 2 weeks (*n* = 62) 2. ER—Daily exposure, plus sticker reward, for 2 weeks (*n* = 60)	No exposure (*n* = 66)	Increased liking at postintervention in I1 and I2 vs C, and no further change over 4-w and 3-m follow-ups. Target V consumption increased postintervention in all groups, and continued to increase for I2 at 4w and 3 m, and for C at 3 m	The findings support the effectiveness of using a reward with a repeated exposure strategy. In particular, such rewards can facilitate the tastings necessary to change liking
Noradilah et al. [147] Exposure	To increase acceptance of a target disliked V in children aged 5–6 years (*n* = 42)	Target V served at lunch on 3 consecutive days	No control	Increased intakes of V in I from day 1 to 3. Parent reported child liking of V also increased	Multiple exposures to V could be a strategy to increase consumption of V among children
Wardle et al. [148] Exposure Rewards	To increase V acceptance in children aged 5–7 years	1. Exposure—8 daily offers to taste and eat target disliked V (*n* = 15) 2. Reward—8 daily offers to taste, eat and gain reward sticker for target disliked V (*n* = 16)	No exposure or reward (*n* = 18)	Increased liking and consumption in I1 vs C. Intermediate effects in I2. Increased intakes in all groups after intervention vs before	Repeated exposure to the taste of unfamiliar V is a promising strategy for promoting liking of previously disliked V in children
Lakkakula et al. [149] Exposure	To increase liking for target V in fourth/fifth grade children (*n* = 360). Part of a wider school-based intervention	Offered cold carrots, tomatoes and bell peppers, and hot peas to taste once a week for 10 weeks	No control	For children who began the programme disliking the target V, I improved liking scores for carrots, tomatoes and peas; liking for bell peppers did not change. For children who began the study liking the target V, no changes were found	Repeated tasting of less-liked vegetables by children in a cafeteria-based setting is a strategy to promote liking of these items, that is effective in approximately half of the participants
Johnston et al. [150] Conditioning	To increase V consumption and V variety in 6th grade children	V paired with a preferred taste (peanut butter) weekly for 4 months (*n* = 40)	V exposure weekly for 4 months (*n* = 38)	Significant increases in vegetable consumption, and variety of vegetables eaten in I vs C	Pairing of vegetables with a preferred taste may be an effective technique for increasing consumption
Zeinstra et al. [151] Conditioning	To increase V preferences and consumption in children aged 7–8 years (*n* = 19)	Seven daily exposures to V juice paired with high energy	Seven daily exposures to V juice paired with low energy	No differences between I and C, but consumption was very low	The pure taste of vegetables is not acceptable enough to allow adequate consumption for flavour-nutrient conditioning to occur
Olsen et al. [152] Exposure Conditioning	To increase V intakes in children aged 9–11 years	1. Neutral V paired with liked V for 6 exposures, followed by 6 exposures to 3 V (*n* = 72) 2. Neutral V alone for 6 exposures, followed by 6 exposures to 3 V (*n* = 74) 3. Neutral V paired with disliked V for 6 exposures followed by 6 exposures to 3 V (*n* = 73)	No exposure	Increases in neutral V intake between I1 vs I2 and I1 vs I3. No other differences. No differences between conditions in liking	Pairing with a liked V increased neutral V consumption. Serving V that are mixed in this manner has potential for increasing intakes

Interventions ordered by age of target audience

*C* comparison, *I* intervention, *V* vegetable, *vs* versus, *w* weeks, *m* months

While largely successful, particularly over considerable exposures, exposure, however, is a relatively time-consuming practice that results in small changes, and typically only for the vegetable to which children have been exposed. Nine interventions have extended the use of exposure to consider exposure to vegetables via picture books and stories (Table 3). These procedures appear beneficial, although few studies have currently tested these ideas, and effects again appear small or unreliable, and typically only apply to the vegetable to which the child has been exposed. Little evidence suggests that effects generalise to other vegetables, so neither taste or visual exposure appears to encourage consumption of a variety of vegetables. Repeated exposure to many vegetables may result in increased consumption of many vegetables, and some studies are beginning to demonstrate these effects [156, 157], but generalisation of exposure to non-exposed vegetables has not yet been demonstrated reliably either through the use of taste or visual stimuli. The potential for exposure to multiple as opposed to single vegetables at one time point, however, may be greater using visual as opposed to taste stimuli.

**Table 3:** Published interventions utilising exposure to picture books containing vegetables to increase vegetable intake

Reference/intervention	Aim	Intervention	Comparison	Results	Conclusions
Heath et al. [153] Exposure via picture books	To increase familiarity and liking for V in 20- to 24-month-old children	Reading a picture book every day for 2 weeks including: 1. a liked V (*n* = 19) 2. a disliked V (*n* = 19) 3. an unfamiliar V (*n* = 19)	Test procedures conducted on target V and control (non-exposed) V	Increased intakes, and reduced encouragement to try unfamiliar V in all I, and particularly I3 vs C. No effects in willingness to taste	Results confirm the potential for picture books to play a positive role in encouraging healthy eating in young children
Bryne and Nitzke [154] Exposure via picture books	To improve attitudes and behaviours towards an unfamiliar V in children aged 3–5 years	1. Childrens book with positive messages about V (kohlrabi) 2. Childrens book with negative messages about V (kohlrabi) (*n* = 118)	No book	More V tasters in I1 vs C during the second posttest	Children’s books with positive messages can increase willingness to taste
De Droog et al. [155] Exposure via picture books Social activities	To increase carrot consumption in children aged 4–6 years	5 days exposure to 1. Picture book containing carrots and rabbit, passive reading (*n* = 26) 2. Picture book containing carrots and rabbit, active reading (*n* = 26) 3. Picture book containing carrots and turtle, passive reading (*n* = 26) 4. Picture book containing carrots and turtle, active reading (*n* = 26)	No exposure to book (*n* = 56)	More carrots consumed in all I vs C. Greater consumption with active v passive reading	Young children seem to enjoy this interactive shared reading, triggering positive feelings that increase children’s liking and consumption of the food promoted in the book

Interventions ordered by age of target audience

*C* comparison, *I* intervention, *V* vegetable, *vs* versus

### Interventions based on changing the environment

An alternative approach to encourage vegetable consumption focuses on changing the environment and increasing consumption through increasing the provision of vegetables, or improving the manner in which provision is implemented (Table 4). Thirty-nine interventions were found using these strategies. All of these, with the exception of three interventions (one increasing variety [158] and two improving presentation [141, 164]) resulted in increased selection and/or consumption of vegetables in children and adults, through the increased provision of vegetables (*n* = 20), through the increased provision of a variety of vegetables (*n* = 7), through improved presentation (*n* = 5), through changing the location of vegetables (*n* = 1), through changing the order in which vegetables and other foods are served (*n* = 1) and through changing the serving order, while also increasing availability (*n* = 2). Increased consumption as a result of increased provision is unsurprising, but concerns have been raised regarding increased energy intakes as a result, and increased potential for food wastage. An absence of effects on overall energy intakes is reported in some studies [161], and concerns are mitigated if vegetables are substituted for other foods in the meal as opposed to simply added [161]. Suggestions to reduce potential food wastage include the use of family style serving dishes for individual meals [159, 161] or allowing differential selection, but again the cost-effectiveness of interventions that can increase waste will be questioned. Strategies that improve the presentation of vegetables may offer a valuable alternative. These interventions typically change the salience or likely appeal of vegetables [141, 164, 166], and have again demonstrated success, but relatively few studies are currently available. Exact mechanisms are again unclear—attractive labels may rely partly on modelling, effects due to serving order may rely partly on hunger and exposure, but the relative ease and low cost of these interventions add to their value.

**Table 4:** Published interventions using increased availability and variety of provided vegetables, and improved presentation of vegetables to increase vegetable intakes

Reference/intervention	Aim	Intervention	Comparison	Results	Conclusions
Coulthard et al. [156] Variety	To increase V intakes in children aged 4–6 months	Exposure to a variety of V over 9 days	Exposure to 1 V over 9 days	Those weaned later (5–6 m) in I consumed significantly more novel V vs C. No effects in those weaned earlier (4–5 m)	Infants who are weaned at 6 m may benefit from being weaned onto a variety of tastes
Maier et al. [157] Variety	To increase novel V acceptance in children aged 7 months	Phase 1 1. 3 daily exposures to 3 V (*n* = 46) 2. 3 alternated daily exposures to 3 V (*n* = 51) Phase 2 All groups given 5 alternate exposures to 2 V	Phase 1 9 daily exposures to 1 V (*n* = 51) Phase 2 5 alternate exposures to 2 V	Intakes and liking of novel V after phase 1 and phase 2 increased in I1 and I2 vs C, greater increases for I2	High variety produced greatest new food intake
De Wild et al. [158] Variety	To increase V intakes in children aged 2–5 years. Home-based intervention	Exposure over 12 meals to 2 target V simultaneously (*n* = 34)	Exposure over 12 meals to 1 target V (*n* = 36)	I positively associated with higher intake than C but not significantly so	Choice-offering has some, but not a robust effect on increasing V intake in children
Spill et al. [159] Availability	To increase V consumption (and decrease energy intake) in children aged 3–5 years (*n* = 51)	Lunch starter provided of: 1. 30 g carrots 2. 60 g carrots 3. 90 g carrots	No lunch starter	Greater target V and total V consumption in all I vs C, and in I2 and I3 vs I1. No differences in total energy intake	Increasing the portion size of a V served as a first course can be an effective strategy for increasing V intakes in preschool children
Spill et al. [160] Availability	To increase V intakes in children aged 3–5 years (*n* = 72)	Lunch starter provided of: 1. 150 g tomato soup 2. 225 g tomato soup 3. 300 g tomato soup	No soup served	Greater intakes of V in I1, I2 and I3 vs C. Increasing the soup portion size increased soup and V intake	Serving low-energy-dense V soup as a first course is an effective strategy to increase V consumption at the meal
Spill et al. [161] Availability	To increase V (and reduce energy) intakes in children aged 3–5 years (*n* = 40)	1. Pureed V (triple content) added to foods across 1 day to reduce energy density by 85 % 2. Pureed V (quadruple content) added to foods across 1 day to reduce energy density by 75 %	Energy density—100 %	V intake increased in I1 and I2 vs C. No compensatory effects on V side dish consumption	The incorporation of substantial amounts of puréed V is an effective strategy to increase daily V intake and decrease energy intake in young children
Correia et al. [141] Presentation	To increase V intakes and willingness to try in preschool children (3–5 years)	Snack: Target V arranged in appealing manner (*n* = 42)	Snack: Target V not arranged in appealing manner	No effects of I	Further research should explore the strategy of pairing vegetables with liked foods, no effects of appearance
Mathias et al. [162] Availability	Intervention to increase V intakes in children aged 4–6 years	Serving of 150 g V at a single meal	Serving of 75 g V at a single meal	Increased V intake in I vs C. Effects limited to those who liked V	Serving larger V portions at meals can be used to promote young children’s intake of V without influencing fruit or total energy intake
Bucher et al. [163] Variety	To increase V intakes in children aged 7–10 years	Selection from buffet (fake foods) of pasta, chicken and 2 V (carrots, beans) (*n* = 34)	Selection from buffet containing pasta, chicken and 1 V (carrots (*n* = 32) or beans (*n* = 34))	Children served themselves more energy from V in I vs C. No differences in total meal energy	Variety is effective in increasing the V choice of school-aged children. Serving an assortment of V in school cafeterias might be a simple and effective strategy to improve children’s nutrition
Morizet et al. [124] Presentation	To increase vegetable dish selection in 8- to 11-year-olds. School-based intervention	Novel V dishes with: 2. basic label (carrots *n* = 44, broccoli *n* = 72) 3. model-related label (carrots *n* = 41, broccoli *n* = 90)	Novel V dishes with no label	Increased intakes in I2 and I3 vs C [I reverses reduced selection for no label (I1, see Table 1)], and no differences between I2 and I3	Adding a label with the V name can increase children’s willingness to select a new V dish instead of a familiar one
Wansink et al. [164] Study 1 Presentation	To increase V selection in children aged 8–11 years old. School-based intervention	1. V served at lunch with attractive label (*n* = 32) 2. V served at lunch with generic label (*n* = 38)	V served at lunch with no label (*n* = 45)	Increased intakes of V in I1 vs I2 and C. No effects on selection	Attractive names effectively and persistently increased healthy food consumption in elementary schools
Wansink et al. [164] Study 2 Presentation	To increase V selection in children aged 8–11 years old. School-based intervention	V served with attractive name every day for 1 month (*n* = 742)	V served with no name every day for 1 month (*n* = 810)	Increased selection of V in I vs C	Attractive names effectively and persistently increased healthy food consumption in elementary schools
Just and Wansink [165] Availability	To increase V selection in a school canteen	Introduction of a salad bar	No salad bar	Increase in number of children consuming salad in I vs C	Simple changes can increase selection
Just and Wansink [165] Variety	To increase V intakes in a school canteen	Giving students a choice of 1 of 2 V	No choice—requiring students to take 1 V	Increased intakes of V in I vs C	Providing choice within forced selection improved intakes
Just and Wansink [165] Location	To increase V selection in a school canteen	Salad bar positioned, so that children must walk round it	Salad bar positioned, to the side	Immediate increase in sales of salad following I vs C. Continued to increase	Simple changes can increase selection
Redden et al. [166] Study 1 Availability Serving order	To increase V intakes in 5th grade children	Mildly liked V provided in isolation while waiting for lunch (*n* = 755). V also provided for lunch	V only provided for lunch (*n* = 680)	Increased total V consumption in I vs C	Intervention increased target V consumption
Redden et al. [166] Study 2 Availability Serving order	To increase V intakes in 5th grade children	Mildly liked V provided in isolation while waiting for lunch (*n* = 486–530). V and control V also provided for lunch	V and control V only provided for lunch (*n* = 529–558)	Increased mildly liked V intake and total intake in I vs C. No change in control V consumption. Sustained effects over 3 days	Intervention increased target V consumption, and did not decrease other V consumption
Reicks et al. [167] Presentation	To increase V intakes in elementary school children (kindergarten—5th grade) (*n* = approx.. 800)	Photographs of target V (carrots, green beans) were placed in lunch trays at one lunch	No photographs, same lunch served	Increased selection of V in I vs C, so increased consumption. Students selecting beans consumed the same in I and C, but students selecting carrots also consumed more in I vs C	Placing photographs in cafeteria lunch trays incurs minimal costs, but was associated with an increase in V consumption within the range of those found in more expensive interventions
Bucher et al. [168] Variety	To enhance V choices and improve meal composition in college students	Buffet meal (fake food) of pasta, chicken and 2 V (carrots, beans) (*n* = 34)	Buffet meal (fake food) of pasta, chicken and 1 V (carrots (*n* = 29) or beans (*n* = 35))	Participants in I chose more energy and more % energy from V vs C. No differences in total energy selected	Serving an assortment of V might be a simple and effective strategy to increase V intakes and improve meal composition
Blatt et al. [169] Availability	To increase V (and reduce energy) intakes in adults (*n* = 41)	1. Pureed V (triple content) added to foods across 1 day to reduce energy density by 85 % 2. Pureed V (4.5 times content) added to foods across 1 day to reduce energy density by 75 %	Energy density—100 %	V intake increased and energy density decreased in I1 and 2 vs C	Large amounts of puréed V can be incorporated into foods to increase V intakes and reduce energy intakes
Meengs et al. [170] Variety	To promote V intakes in adults (*n* = 66)	1 meal involving 200 g each of 3 V	Three meals involving 600 g of 1 V (same three V)	Increased V intake at I vs C. Increased V intake at I vs most preferred C	Increasing the variety of V served at a meal can be used to increase V intake
Redden et al. [166] Study 3 Serving order	To increase V intakes in adults	Mildly liked V provided in advance of other more liked foods (*n* = 36)	Other foods provided: 1. in advance of V (*n* = 43) 2. simultaneously (*n* = 39)	More V consumed in I vs C1 and C2	Eating V first in isolation may prove useful for increasing V consumption in a wide range of individuals
Rolls et al. [171] Availability	To increase V intakes (and facilitate weight management) in adults	Addition Study (*n* = 49) 1. 270 g V served 2. 360 g V served Other meal components unchanged Substitution Study (*n* = 48) 1. 270 g V served 2. 360 g V served Other meal components reduced proportionally	Addition Study 180 g V served Other meal components served Substitution Study 180 g V served Other meal components served	Greater V served led to greater V consumed in both studies Effects on meal energy density and energy intake also in the substitution study	Serving more V, either by adding more or substituting them for other foods, is an effective strategy to increase V intake at a meal
Shenoy et al. [172] Availability	To increase V intakes (and improve CVD health) in healthy adults	Education on the DASH diet and: 1. 8 oz V juice daily (*n* = 30) 2. 16 oz V juice daily (*n* = 30)	Education on the DASH diet only (*n* = 30)	I1 and I2 increased V intakes (and improved micronutrient profiles) vs C (and decreased blood pressure in prehypertensive adults)	Including 1–2 cups of vegetable juice daily was an effective and acceptable way for healthy adults to consume more V
Shenoy et al. [173] Availability	To increase V intakes (and improve CVD health) in adults with metabolic syndrome	Education on the DASH diet and: 1. 8 oz V juice daily (*n* = 27) 2. 16 oz V juice daily (*n* = 27)	Education on the DASH diet only (*n* = 27)	I1 and I2 increased V intakes vs C (and decreased blood pressure. No effects on CVD measures)	Including 1–2 cups of vegetable juice daily was an effective and acceptable way for healthy adults to consume more V

Interventions ordered by age of target audience

*C* comparison, *I* intervention, *V* vegetable, *vs* versus

### Interventions based on changing or using cognitive factors

Nineteen interventions were found that used information, education or other cognitive techniques (Table 5). These interventions are largely aimed at older audiences (those where cognitive factors have a greater impact on vegetable consumption and non-consumption), and used a range of techniques from providing information and education on nutrition (*n* = 6) or nutrition-related skills (*n* = 2), providing education plus a demonstration (*n* = 1) or gardening experience (*n* = 2), providing tailored information (*n* = 2), providing information on social norms (*n* = 1), invoking choice (*n* = 4) and invoking a memory (*n* = 1). With the exception of one intervention that aimed to educate [75], and two interventions that utilised choice [174], all of the studies using these types of strategy reported success to some degree, but multiple measures of impact were often taken, and success is not necessarily reported for all measures. The cost-effectiveness of these types of interventions is, however, also often questioned. Educational interventions can be costly, particularly those involving classes or courses to be delivered by a professional, but the long-term benefit of these interventions can also be difficult to assess. Knowledge accumulates over time and experience, and it can be difficult for individuals to pinpoint the exact source/sources of beneficial education.

**Table 5:** Published interventions using information provision, education and other cognitive strategies to increase vegetable intakes

Reference/intervention	Aim	Intervention	Comparison	Results	Conclusions
Wardle et al. [75] Education	To increase liking and intake for a previously disliked V in children aged 2–6 years. Home-based intervention	Information—nutritional advice and leaflet (*n* = 48)	No intervention (wait-list) (*n* = 45)	No differences between I and C	A parent-led, exposure-based intervention involving daily tasting of a V holds promise for improving children’s acceptance of to increasing liking for a previously disliked V. No effects for information only
Zeinstra et al. [174] Choice	Intervention to increase V intakes in children aged 4–6 years. Single restaurant meal intervention	1. Pre-meal choice—Single choice at the start of the meal of 1 of 2 V (*n* = 110) 2. At-meal choice—Repeated choices throughout the meal of 2 V (*n* = 97)	No choice—provision of 1 of 2 V (*n* = 96)	No differences in V liking or intake between conditions. Some effects of individual differences	Having a pre-meal choice was appreciated by the children but did not affect intake, liking, or motivation to eat vegetables
Dominguez et al. [175] Choice	Intervention to increase V intakes in children aged 4–6 years	1. Single choice at the start of the meal of choice of 2 V (*n* = 50) 2. Repeated choices throughout the meal of choice of 2 V (*n* = 56)	No choice—provision of 2 V (*n* = 44)	Total V intakes were higher in I1 and I2 vs C	Results demonstrate the enhancing effect of providing choice to increase V intakes in young children
Gholami et al. [176] Skills-based education	To increase V provision and consumption in 6–11 year old children	Theory based instructional leaflets to promote self-regulatory skills for providing healthy nutrition for children	No intervention (usual practice)	Increased V intake in I vs C, 2 weeks postintervention. No difference 3 months postintervention	Engaging mothers in self-regulatory health promotion programmes may facilitate more vegetable intake among their daughters
Morris and Zidenberg-Cherr [177] Education Exposure Experience	To increase V intakes in 9–10 year olds (4th grade). School-based intervention	1.NL—nutrition education (*n* = 71 in school 1) 2. NG—nutrition education plus gardening activities (6 V planted) (*n* = 81 in school 2)	No formal nutrition or gardening education (*n* = 61 in school 3)	Knowledge and preferences for 2 V, increased, in I1 and I2 vs C, postintervention, and remained for 1 V per I at 6 months. Preferences for 1 V and 1 additional (non-planted) V increased in I2 vs I1 and C, postintervention and at 6 months. No differences in willingness to taste V	Garden-enhanced nutrition education is an effective tool for improving nutritional knowledge and V preferences
Morgan et al. [178] Education Experience	To increase V intakes, V preferences and FV knowledge in 11- to 12-year-olds. School-based intervention	1. NE—10 week nutrition education (*n* = 35) 2. NEG—10 week nutrition education and garden (*n* = 35)	No intervention (wait list) (*n* = 57)	Greater willingness to taste V and greater taste ratings for I1 and I2 vs C. No differences in V intakes	School gardens can impact positively on willingness to taste V and V ratings, but more comprehensive strategies are required to increase V intakes
Robinson et al. [179] Study 3 Memory	To increase V intakes in university students	Recall positive V memory	Recall of other memories	Increased V intake in I vs C	Recall of previous eating experiences could be a potential strategy for altering food choices
Stok et al. [180] Study 1 Education	To increase V intakes in university students	Descriptive social norm—majority norm	Descriptive social norm—minority norm	Marginally significant increase in V intakes in I vs C	A norm describing the behaviour of a salient social group can impact on behaviour
Ogawa et al. [181] Education	To increase V purchasing behaviour in adults	Point of purchase (POP) health information for V presented in supermarket store for 60 days	Control store (same supermarket chain)—no information	Increased sales at I vs C. Adjustments made for seasonal effects and number of customers	Health-related POP information for V in supermarkets can encourage customers to purchase V
Rahman et al. [182] Education	To increase dark leafy green vegetable (DLGV) presentation to children aged 6–35 months by mothers	1. health education, plus feeding demonstration (*n* = 44) 2. health education only (*n* = 36)	No intervention (usual practice) (*n* = 80)	Increased number of mothers presented DLGV at an impromptu meal 8 weeks later in I1 and 2 vs C Maternal literacy and family income controlled for	Education for mothers was effective at increasing DLGV intakes in children
Tabak et al. [183] Education	To increase presentation of V by mothers, and V intakes in children aged 2–5 years	Parents sent 4 tailored newsletters and given 2 motivational phone calls over 4 months (*n* = 22)	Parents sent 4 children’s books (1/month) (*n* = 21)	Increased availability and offering of V in I vs C. No differences in intakes	Home-based interventions to alter parental feeding practices and the home environment may help towards increasing V intake in children
Wenrich et al. [105] Skills-based education	To increase serving and consumption of deep-orange, cruciferous and DGLV in families	8 weekly interactive sessions for food preparers, including recipes and handouts	8 weekly mailings that included similar recipes and handouts	No differences in servings or intakes between I vs C, at end of intervention or three-month follow-up. More recipe use by I vs C	Tools to help the food preparer draw family members into recipe evaluation are useful
Clarke et al. [184] Education	To increase V intakes in clients of community pantries	1. Tailored tips and recipes (*n* = 244) 2. Generic tips and recipes (*n* = 226)	No tips or recipes (*n* = 236)	Increased V use in I1 vs I2 and C	Results demonstrated benefits of tailoring over both generic and control conditions

Interventions ordered by type and age of target audience

*C* comparison, *I* intervention, *V* vegetable, *vs* versus

### Multi-component interventions

Multi-component interventions involve a combination of strategies (Table 6). Ten of these interventions were found. Again all the published reports evaluating these interventions report success, but again multiple measures are often taken, which demonstrate varying degrees of benefit. These types of intervention can also be time-consuming and costly to implement. Success is furthermore not easily attributable to the combination of many strategies as opposed to the use of any single one.

**Table 6:** Published multi-component interventions using a variety of strategies to increase vegetable intakes

Reference/intervention	Aim	Intervention	Comparison	Results	Authors’ conclusions
Faber et al. [185] Education Availability Exposure Experience	To improve intakes of yellow and dark green leafy V (DGLV) in children aged 2–5 years. A rural home gardening intervention	Home gardening programme in a rural village, as part of a primary care activity (*n* = 126 home gardens, 1/3 households)	Neighbouring village with no home gardening programme	At 20-month follow-up, children from I consumed yellow and DGLV more often vs C. Maternal knowledge also improved in I	A home gardening programme that was integrated with a primary health care activity, linked to nutrition education, and focused on the production of yellow and DGLV improved the vitamin A status of 2- to 5-y-old children in a rural village in South Africa
Bai et al. [186] Education Exposure Rewards Experience	To increase attitudes, intentions and V consumption. School-based intervention in children in US third grade	Nutrition education, poster displays, featured V in canteen (1/month for 9 months), and daily V tasting (4 preparations) (*n* = 38)	No intervention (usual practice) (*n* = 35)	Self-report V intakes higher in I vs C. Attitudes predicted consumption in I, social norms predicted consumption in C. Schools matched for race and gender profiles	The Veggiecation programme generated a positive attitude to influence vegetable intake in school
Leak et al. [187] (protocol only)	To increase V intake, liking and variety in children aged 9–12 years. Intervention for caregivers	Intervention based on 9 behavioural economics strategies for 6 weeks (*n* = 36)	Usual practice for 6 weeks (*n* = 10)	Protocol only	Protocol only
Wright et al. [188] Education Exposure Experience	To increase V selection at a salad bar in kindergarten—5th grade children. School-based intervention	Gardening programme for three weeks, including V growing, tasting and consuming, and increasing knowledge	No control	V selection increased during I and continued to rise post I to a lesser extent	Gardening intervention lessons and activities impacted on V intakes
Ratcliffe et al. [189] Education Experience	To increase V intakes in children aged 11–13 years. School-based intervention	Health and science education. Garden based education (*n* = 170, 2 schools)	Health and science education only (*n* = 150)	Increased self-report recognition of, attitudes towards, preferences for, willingness to taste, and V variety consumed in I vs C. No differences in taste test	Gardening improved recognition of, attitudes towards, preferences for, willingness to taste and variety of V eaten
Brown et al. [97] Education Exposure Experience	To increase readiness to change, V self-efficacy and V intakes in college students. College based intervention (*n* = 186)	Online preparation videos and tasting of 4 V, one per month for 4 months (*n* = 186)	No control	Stage of change and V self-efficacy increased postintervention. Intakes for one V increased, but no effects for other V or all V	Online V demonstration videos may be an effective and cost-efficient intervention for increasing self-efficacy of V preparation and readiness to increase V consumption among college students
Carney et al. [190] Education Experience Social activities	To increase V intakes (reduce food security and improve family relationships) in families	Community gardening programme, including education, gardening experiences, and social activities (*n* = 42 families, 163 individuals)	No control	I increased V intakes (reduced food insecurity and improved family relationships) from pre- to postintervention	A community gardening programme can increase V intakes, reduce food insecurity and improve family relationships
Schreinemachers et al. [191] Availability Education Experience	To increase V production, consumption and dietary diversity in families	Garden training, education, seeds, cooking, and garden equipment (*n* = 103)	No intervention (wait-list) (*n* = 479)	I resulted in increased V production, consumption and dietary variety vs C	Women’s home gardens are an effective intervention for increasing supply and consumption of a range of V in poor households, and so contributing to nutrition security
Kushida and Murayama [192] Availability Education	To increase V consumption behaviours in adults in workplace cafeterias	12 informational table tents placed every 2 weeks on all cafeteria tables, posters and locally grown V included in cafeteria menu. Personal dietary feedback for all participants (*n* = 181)	No intervention, Personal dietary feedback for all participants (*n* = 168)	Increased V consumption in I vs C in the cafeteria, and across the day (self-report)	Findings suggest a beneficial effect of providing access to nutrition information about V consumption
Weatherly and Weatherly [193] Availability Education Experience	To increase V consumption (and increased interaction and self-worth) in homebound adults	Container V garden	No control	Reports of improved interaction and self-worth. Participants received fresh produce, but no results provided for V consumption	The containerised V garden programme has many benefits, for homebound adults and volunteer helpers

Interventions ordered by age of target audience

*C* comparison, *I* intervention, *V* vegetable, *vs* versus

## Discussion

While a variety of successful strategies for increasing vegetable intakes have been tried, evaluated and published, evaluation periods are typically short, effect sizes can be small, and those studies that use longer follow-up periods often report reductions in effect size as follow-up periods are extended. These findings are unsurprising and have persuaded many researchers to recommend repeated interventions or a combination of interventions with the hope of improving long-term benefits. However, cost-effectiveness is rarely considered, yet cost-effectiveness becomes an increasing concern in long-lasting and multi-component interventions. Further work is clearly still required. A greater number and variety of intervention evaluations would increase the evidence base, and more reliably inform future policies. Longer-term follow-ups for interventions are imperative, and consideration of more sustainable behaviours or the more sustainable elements of behaviour, such as habit formation or behavioural norm changes, would be of value.

While the review highlights strategies of benefit furthermore, the review also identified noticeable absences. Based on the search strategies and current literature, very few interventions were identified specifically for adolescents or older individuals. The eating attitudes, practices and intakes of these groups are known to differ from those of other members of the population, and the simple generalisation of successful strategies from other population groups may not occur. Adolescence is a period of rapid development, from physical, cognitive and social perspectives, and changes to eating practices and dietary intake during this period are well reported [194]. Studies in this group on barriers to consumption identify constraints largely similar to those in younger children, but also identify an increased recognition of cognitive factors. Strategies then that involve education may be particularly beneficial. Older individuals similarly will experience changes in physical and cognitive abilities, many of which will have an impact on eating practices and food intake [195–197]. Barriers specifically to vegetable consumption in this group have not been identified as far as we are aware, but barriers to fruit and vegetable consumption are similar to those for other adults [195, 196], although the impact of demographic characteristics and environmental factors tend to be exacerbated. Changes to living circumstances for example, will impact negatively on existing impacts as a result of availability, cost and cooking abilities [195–197].

Vegetable consumption is also known to be low in individuals of low education and of low socio-economic status [198], and these factors are specifically highlighted as barriers to increasing consumption, yet few of the interventions published to date focus on or even include individuals with these demographics. There are some exceptions—the intervention by Clarke et al. [184] focuses specifically on individuals using community pantries, and many of the studies in the developing world focus not only on increasing vegetable intakes but also on sustainable vegetable provision and improved food security [185, 191], but more work is clearly needed in relation to socio-economic disparities. Interventions that improve fruit and vegetable intakes are available [199]. Increased efforts to reduce socio-economic disparities, however, are often requested [200–203], and concerns that intervention success is most easily achieved in those of little need of benefit are difficult to allay.

Consideration of the barriers to vegetable consumption suggests that many of the strategies that have shown success so far in certain groups may be beneficial for other groups. Almost all individuals will arguably benefit from increasing vegetable intakes, and the strategies found to be successful in one population group may easily transfer to another. Exposure type strategies to increase liking, for example, have shown effects for fruit consumption in older adults as well as children [204], although vegetables were not specifically investigated here. We recommend careful consideration of barriers however, and caution against a ‘one size fits all’ approach. While interventions may be successful across individual and population groups, testing is clearly required. At present, there is a real lack of comparisons between interventions—i.e. interventions have not been compared, e.g. in the same age group or population, with the exception of comparisons of differing exposure and conditioning strategies in young children. This lack of comparison may reflect the early nature of the field, but even where multi-component interventions have been successful, identification of the successful component/components is rarely undertaken. With a view to lasting impact and cost-effectiveness, comparison of interventions, or the identification of more effective intervention components would clearly be of value.

Several types of broader population-based interventions have also not yet been considered specifically for vegetable consumption. Strategies such as pricing and marketing interventions, improved product provision, government subsidies, and population-wide awareness and education campaigns [205–207] specifically for vegetables do not yet exist or have not yet been evaluated as far as we are aware. In some countries, WHO recommendations have been separated for fruit and vegetables. Dutch consumers are asked to consume 2 + 2 (2 portions of fruit and 2 portions of vegetables per day), and Australian consumers are asked to aim for 2 + 5 (2 portions of fruit and 5 portion of vegetables per day), but these types of recommendations rely heavily on an individual’s ability to identify and categorise fruits and vegetables, and limited work suggests that consumers find this difficult [41, 104].

### Limitations of the review

While our review has identified a number and variety of interventions, we have only considered the published literature, and our search strategy is likely to be biased towards articles published in English and away from related grey literature, such as lay publications and conference proceedings. Due to the early nature of the research area, publication bias is also highly likely. There is a noticeable absence of publications that report failures, or that demonstrate cost-inefficiency or other negative impacts of interventions. We also chose not to attempt to combine interventions. Due to the early nature of the research area, the limited number of studies available per intervention type, and high heterogeneity between study methodologies, we considered combination to be inappropriate.

### Future directions

There is an urgent need for the development and evaluation of interventions to target all population groups. Interventions are particularly required for certain population groups, including adolescents, older adults and those of low socio-economic status. Interventions for groups ‘at risk’ of disease may also be beneficial, given the often increased success of interventions in these individuals [1]. Assessments of the long-term benefits, sustainability and cost-effectiveness of interventions are also clearly required. While many interventions report success, effect sizes are typically small, long-term follow-up is rarely undertaken and studies that do report follow-up often fail to find sustained benefits. Interventions with a focus on long-term benefits and sustained behaviour change are required, as is increased work understanding the principles underlying behaviour change and behaviour change maintenance. Comparisons between interventions, to identify those of greatest likely benefit, would also be of interest.

## Conclusion

In conclusion, increasing evidence suggests health benefits from the increased consumption specifically of vegetables, yet barriers to increasing intakes are prevalent, and while successful interventions have been published, the true value of these, both in cost-efficiency and sustainability are yet to be determined. Considerable further work is needed in developing new and adapting existing interventions for all population groups, and in evaluating benefit and cost-efficiency over the longer term.

## Abbreviations

C: Comparison
I: Intervention
V: Vegetable
vs: Versus
w: Weeks
m: Months
